# Reply to “When Is a Ketogenic Diet Ketogenic? Comment on Satiating Effect of a Ketogenic Diet and Its Impact on Muscle Improvement and Oxidation State in Multiple Sclerosis Patients. *Nutrients* 2019, *11*, 1156”

**DOI:** 10.3390/nu11081919

**Published:** 2019-08-15

**Authors:** María Benlloch, María Mar López-Rodríguez, María Cuerda-Ballester, Eraci Drehmer, Sandra Carrera, Jose Joaquin Ceron, Asta Tvarijonaviciute, Javier Chirivella, David Fernández-García, Jose Enrique de la Rubia Ortí

**Affiliations:** 1 Department of Nursing, Catholic University San Vicente Mártir, 46001 Valencia, Spain; 2 Department of Nursing, University of Almería, 04120 Almería, Spain; 3 Department of Physical Activity and Sports Sciences, Catholic University San Vicente Mártir, 46001 Valencia, Spain; 4 Department of Health Sciences, Catholic University San Vicente Mártir, 46001 Valencia, Spain; 5 Interdisciplinary Laboratory of Clinical Analysis, Campus of Excellence Mare Nostrum, University of Murcia, 30100 Murcia, Spain; 6 Fundación FIVAN, 46005 Valencia, Spain

In response to the questions raised in the comment to the editor of R.J. Klement [1], the authors of “Satiating Effect of a Ketogenic Diet and Its Impact on Muscle Improvement and Oxidation State in Multiple Sclerosis Patients” [2] would like to list the following aspects.

1. As Dr. Klement indicates, the diet prescribed to the patients in this study does not fit the definition of “ketogenic diet” as such, since it is characterized by a total carbohydrateintake of lessthan 50 g per day and a moderateproteinintake of approximately 1.5 g per day per kg of referenceweight [3,4]. Therefore, we agree that it would be more correct to change the title to “Satiating Effect of Coconut Oil-Enriched Mediterranean Diet and Its Impact on Muscle Improvement and Oxidation State in Multiple Sclerosis Patients”.

2. Our goal was to administer coconut oilinthe diet as the main source of fat, since it is a substance that presents a large amount of medium-chain fatty acids (MCFAs), which are absorbed and metabolized more quickly and efficiently than long-chain fatty acids (LCFAs) [5]. Furthermore, as previously stated, through the administration of this oil, a ketogenic state is produced with respect to the beginning of the study, asthe ketones representan alternative brain fuel to glucose [6]. Moreover, it has been shown that the intake of coconut oil may improve brain health by directly activating ketogenesis in astrocytes and, thereby by providing fuel to neighboring neurons [7]. This fact means that, regardless of the ketone values in blood, coconut oil is the best alternative in order to reach this brain ketogenesis, with enormous resulting potential benefits in neurodegenerative diseases in general, and in MS in particular in neurodegenerative diseases in general, and in Multiple Sclerosis (MS) in particular. In addition, our amounts of coconut oil were very high (60 mL/day divided into two 30 mL doses), so we believe that our results are not comparable to those in the reference indicated by Dr. Klement, where 20 mL were administered daily.

It should also be taken into account that our main objective was to see the improvements achieved with our diet, comparing the variables measured in the study before and 4 months after the intervention. For this, although ketone bodies were not measured postprandially, we were already able to see significant differences in ketone bodies before and after the intervention, which correlated with the significant change in satiation (expected with the elevation of ketone bodies in blood). This fact, together with the significant anthropometric improvements, suggests that the diet could be considered that have positive effects for these patients, and that it fulfilled the objectives of the study.

3. As Dr. Klement indicated, the patients were advised to eat five meals a day. This way of providing the nutrients is healthier in this type of patients, since, after analyzing their eating habits, it was determined that most of them ate in a disorganized way and had only a few meals, which were typically based mainly on simple carbohydrates. This is the reason why, despite the five daily meals, it was possible to significantly increase the concentration of ketone bodies in the blood, thus improving satiation.

4. We believe that a fasting increase of almost double the blood concentration of the ketone body beta-hydroxybutyrate (BHB) indicates ketogenesis with respect to the same group before changing their diet. In addition, the fact that BHB was measured on an empty stomach, despite having been measured after a meal in other studies, suggests that these levels were comparatively much higher throughout the day than before the patients received the treatment. This indicates that there was a sustained (and therefore more effective) elevated level of BHB, which allowed the aforementioned improvements to be achieved in the patients.

On the other hand, responding to the comment regarding transporters, in Figure 1. A we indicated that MCT1 and MCT4 are ketone body transporters, based on the publications of Hernández et al. [8,9]. 

Finally, we appreciate the information related to the “Spanish Ketogenic Mediterranean Diet” and we will take it into account for future studies, asit is highly interesting. However, we consider that the type of prescribed diet does not fit into the characteristics of the diet in this study, since our main source of fat (as previously discussed) was coconut oil, whereas in the “Spanish Ketogenic Mediterranean Diet” it is olive oil [6].

After reviewing your contributions, we would like to sincerely thank you for all the information and opinions you provided. We appreciate this opportunity to improve of our methodology for future studies, and thus be able to better contribute to scientific knowledge.
